# Transforming growth factor-β1 and eosinophil-derived neurotoxins contribute to the development of work-related respiratory symptoms in bakery workers^☆^

**DOI:** 10.1016/j.waojou.2019.100058

**Published:** 2019-10-01

**Authors:** Hoang Kim Tu Trinh, Bastsetseg Ulambayar, Thi Bich Tra Cao, Eun-Mi Yang, So-Hee Lee, Hae-Sim Park

**Affiliations:** a Department of Allergy and Clinical Immunology, Ajou University Medical Center, Suwon, South Korea; b Department of Biomedical Science, Ajou University School of Medicine, Suwon, South Korea; c Department of Allergy and Clinical Immunology, Department of Biomedical Science, Ajou University School of Medicine, Suwon, South Korea

**Keywords:** Bakery workers, Work-related symptoms, Transforming growth factor β1, Eosinophil-derived neurotoxin

## Abstract

**Background:**

In baker's asthma previous studies suggest that adaptive and innate immunity are involved in the development of work-related respiratory symptoms (WRS), where we hypothesized that epithelial cells derive airway inflammation through modulating the release of inflammatory cytokines. Thus, we conducted this study to investigate the role of epithelial cell-derived cytokines in the development of WRS among bakery workers.

**Methods:**

We recruited 385 wheat-exposed subjects with WRS (WRS+)/without WRS (WRS-) working in a single industry and 243 unexposed controls from Ajou Medical Center (Suwon, South Korea). Levels of epithelial cell-derived cytokines (interleukin [IL-8], transforming growth factor-β1 [TGF-β1], eotaxin-2) and inflammatory mediators (eosinophil-derived neurotoxins [EDN]) in sera or cell-free supernatants were measured by ELISA. Human airway epithelial cells (HAECs), A549, were stimulated by wheat flour extracts and co-cultured with peripheral blood neutrophils isolated from 4 asthmatic patients.

**Results:**

Serum TGF-β1 levels were significantly lower in exposed subjects than in unexposed controls, in the WRS+ group than in the WRS- group (*P* < 0.001 for each). The WRS+ group had a significantly higher level of serum EDN than the WRS- group (*P* < 0.001). Serum TGF-β1 and EDN levels predicted the development of WRS in exposed subjects (area under the curve [AUC] = 0.719, 72.4% sensitivity/70% specificity; AUC = 0.759, 78.6% sensitivity/60% specificity). From wheat-stimulated HAECs, TGF-β1 release peaked at 6 hours after wheat exposure, while eotaxin-2 peaked at 12 hours. Co-culture of HAECs with neutrophils did not affect TGF-β1 release.

**Conclusions:**

Our results suggest that TGF-β1 may contribute to develop type-2 airway inflammation and WRS. Serum TGF-β1/EDN levels may be potential serum biomarkers for predicting WRS among bakery workers.

## Introduction

Baker's asthma (BA) is a common phenotype of occupational asthma (OA) worldwide. Working in the bakery is associated with exposure to airborne flour dust during the process of making bakery products which affects the risk of work-related respiratory symptoms (WRS), further developing rhinitis and asthma.^1^, ^2^ Previous studies have reported that the estimated prevalence of OA of adult -onset asthma differs among geographic regions, study designs and populations in exposed subjects (5%–10% in Europe and 10–23% in United States).^3^ Up to 33% of adult asthmatic patients have work-aggravated asthma, in which a delayed diagnosis remains common and results in worse outcomes.^4^, ^5^ Flour is a high molecular weight agent that can cause OA (8.5%), and 17.1% of bakery workers were found to have WRS in the Korean population.^1^, ^6^

OA is a result of multiple environmental and genetic influences in which IgE-dependent and/or non-IgE-dependent immunologic/non-immunologic mechanisms are involved. The major causes of occupational allergens are characterized by molecular weight (high and low molecular weight antigens), exposure route/intensity and epigenetic factors.^7^ When exposed to antigens in the workplace, human airway epithelial cells (HAECs) are the first target to release a variety of pro-inflammatory cytokines and chemokines; loss of its protection properties may enhance innate/adaptive immune responses in the asthmatic airway.^8^

Previous studies have demonstrated that inhalation of wheat flour can induce IgE/IgG-mediated immune responses to develop WRS.^9^, ^10^ In addition, wheat contains bacterial endotoxins and lipopolysaccharides that induce innate immune responses involved in the pathogenesis of asthma.^11^, ^12^ Wheat exposure could induce release of interleukin (IL)-8 from HAECs that enhance neutrophil migration and activation in collaboration with myeloperoxidase (MPO) levels.^13^, ^14^ Increased level of S100A8 and folliculin from HAECs and mannose-binding lectin are involved in innate immune responses in bakery workers,^15^, ^16^, ^17^ where eosinophil activation and their interaction with inflammatory cells have not been established yet. Based on these findings, we hypothesized that epithelial-derived cytokines may contribute to develop type 2 airway inflammation in the development of WRS in bakery workers and evaluated the levels of serum TGF-β1, eosinophil-derived neurotoxins (EDN), and eotaxin-2 in wheat flour-exposed workers in order to further understand its pathogenic mechanisms.

## Materials and methods

### Study subjects

We enrolled 385 bakery workers (occupationally exposed) working in a single large bakery and 243 non-exposed subjects (healthy controls). Age, gender, forced expiratory volume in 1 s (% predicted FEV1) values and serum total IgE levels were obtained. A questionnaire was used to collect histories from bakery workers including exposure duration and the presence of WRS based on whether subjects had upper (nasal itching, runny nose, sneezing or congestion) or lower respiratory symptoms (wheezing, shortness of breath, coughing and sputum secretion) and exacerbation conditions (symptoms worsen in the working environment and improve when away from occupational exposure) as previously described.^18^ Atopy was defined by response to skin prick test (SPT) to common inhalant allergens including a mixture of trees and grass, mugwort, ragweed, cat and dog fur, *Dermatophagoides pteronyssinus*, *Dermatophagoides farinae* (Bencard, Bretford, UK) and considered as positive, if subject has more than one positive response to these inhalant allergens. SPT to wheat flour extract was performed in exposed subjects and a positive response was evaluated by the ratio of the mean wheal diameter of the allergen to histamine ≥1. Serum total IgE was measured by ImmunoCAP system (Thermofisher, Waltham, MA, USA) according to the manufacturer's instructions. Serum specific IgE (sIgE) to wheat flour extract was measured by ELISA as previously described.^1^ Written informed consent was collected from individual study subjects. The study was approved by Ajou University Institutional Board. (AJIRB-GEN-SMP-13-108).

### Measurement of serum cytokines

As epithelial derived cytokines, serum levels of transforming growth factor β1 (TGF-β1, R&D Systems, Minneapolis, MN, USA), eotaxin-2 (RayBio Inc, Norcross, GA, USA) as well as IL-8 (Endogen Inc., Woburn, MA, USA) were measured by ELISA. As inflammatory markers, serum levels of MPO (Biocheck Inc., Förster city, CA, USA) and EDN were measured by ELISA using the K-EDN kit (SKIMS-BIO Co. Seoul, Korea) according to the manufacturer's instructions. Serum samples were collected and frozen at −70 °C and thawed before use.

### Culture of HAECs

HAECs (A549 cells) were obtained from the American Type Culture Collection (Manassas VA, USA). Cells were cultured in the RPMI-1640 medium (Invitrogen, Carlsbad, CA, USA) supplemented with 10% heat-inactivated fetal bovine serum, penicillin (100 IU/mL) and streptomycin (50 μg/mL). Cells were maintained at 37 °C in an atmosphere of 95% humidified air and 5% CO_2_.

### Isolation of peripheral blood neutrophils (PBNs)

Peripheral blood neutrophils (PBNs) from 4 asthmatics were collected into BD Vacutainer ® tubes containing acid citrate dextrose solution (BD Biosciences, Franklin Lakes, NJ, USA). As previously described,^19^ blood was layered onto Lymphoprep™ solution (Axis Shield, Oslo, Norway) and centrifuged at 2000 rpm at 20 °C for 25 min without braking. The layer containing granulocytes and red blood cells (RBCs) was sedimented for 30 min in 2% dextran, diluted in Hank's balance salt solution (HBSS) buffer supplemented with 2 mM ethylenediaminetetraacetic acid (EDTA) at room temperature. The upper layer was harvested and washed once with HBSS buffer supplemented with 2 mM EDTA. The eosinophils contaminated were excluded by using the Eosinophil Isolation Kit and MACS Column (Miltenyi Biotec Inc, Auburn, CA, USA) according to the manufacturer's protocols. Cell viability (>98%) and purity (>95%) were assessed by Trypan blue staining and flow cytometry based on CD11b (neutrophil marker). The isolated PBNs were suspended in the culture medium for 30 min prior to further experiments.

### Wheat stimulation of HAECs

A549 cells (1 × 105 cells) were seeded onto a 12-well plate. Cells were starved with serum-free RPMI prior to stimulation. For a dose-dependent assay, cells were stimulated with different doses of wheat flour extract (1, 10 and 100 μg/mL) for 24 hours. For a time-dependent assay, cells were stimulated with wheat flour extract 10 μg/mL for 3, 6, 12, 24 and 36 hours. For the co-culture assay, we used the relatively high number of PBNs (5 × 105 cells) and high dose of wheat flour extract (100 μg/mL). A549 cells (1 × 105 cells) in the bottom and PBNs (5 × 105 cells) on the top were separated in a trans-well culture (Corning Costar® Transwell, Sigma-Aldrich, St. Louis, MO, USA) with 0.4-μm inserts. Wheat flour extract (100 μg/mL) was added to the A549 layer. Cells were re-suspended in serum-free RPMI for 48 hours, and then cell-free supernatants were collected and stored at −70 °C until further analysis.

### Statistical analysis

All statistical analyses were performed using SPSS statistical software version 25 (SPSS Inc, Chicago, IL, USA). We log-transformed the data of all serum cytokine levels before statistical analysis to correct skewed distribution. Student's *t*-test was used to compare parametric variables and the Mann-Whitney *U* test was used to compare non-parametric variables when data is not normally distributed. The χ^2^ test was used to compare the frequency of categorical variables. Multivariate logistic regression was applied to assess the association between exposure and WRS with IgE and serum cytokines, controlling for age and gender. Diagnostic values of serum cytokines in differentiating WRS+ and WRS- subjects were determined by receiver operating characteristic (ROC) curves. Binary logistic regression analysis was used to predict the development of WRS among exposed subjects. Pearson and Spearman's rank correlation coefficients were used to examine correlations between cytokine levels in serum and in cell-free supernatant. Graphs were made using GraphPad Prism software version 7 (GraphPad Software, San Diego, CA, USA).

## Results

### Characteristics of the study subjects and serum cytokine levels according to wheat exposure

The clinical characteristics of the study subjects based on occupational wheat exposure are summarized in Table 1. Age was not different between exposed and non-exposed subjects, and the proportion of males was higher in exposed subjects (*P* = 0.033). Overall, 35.29% of the exposed subjects were atopic and 18.18% had upper and/or lower respiratory symptoms. The mean duration of occupational exposure to wheat was 3.96 years. The exposed workers with WRS had a significantly higher atopy rate and positive SPT responses to wheat allergen (*P* < 0.001 for each). The serum levels of IL-8 was significantly higher in exposed workers than in unexposed controls (*P* < 0.001), while TGF-β1 levels were significantly lower in exposed workers (Table 1). Although there were no significant differences in serum eotaxin-2 or EDN levels between exposed and unexposed subjects, among the exposed subjects, the serum EDN levels were significantly higher in those with positive SPT responses to wheat allergen (*P* = 0.013) and having atopy (*P* < 0.001) (Table S1).

**Table 1:** Clinical characteristic of the study subjects

	Exposed (N = 385)	Non-exposed (N = 243)	*P* value
Age (yr)	35.05 ± 7.81 (n = 382)	37.20 ± 11.30 (n = 235)	0.109*
Gender (male, %)	218/382 (57.07%)	117/242 (73.14%)	0.033
Atopy (positive, %)	132/374 (35.29%)	NA	NA
Exposure duration (working years)	3.96 ± 3.48	NA	NA
WRS (positive, %)	70 (18.18%)	NA	NA
FEV_1_ (% predicted)	94.81 ± 12.62 (n = 379)	NA	NA
SPT to wheat (positive, %)	25/375 (6.67%)	NA	NA
Serum total IgE (IU/L)	1.91 ± 0.64 (n = 377)	NA	NA
sIgE to Wheat (positive, %)	22/367 (5.99%)	NA	NA
IL-8 (pg/mL)	2.14 ± 0.59 (n = 376)	1.33 ± 0.40 (n = 239)	<0.001
MPO (ng/mL)	2.08 ± 0.24 (n = 376)	2.21 ± 0.34 (n = 230)	<0.001
TGF-β1 (pg/mL)	1.45 ± 0.13 (n = 385)	1.50 ± 0.18 (n = 243)	<0.001
EDN (ng/mL)	1.57 ± 0.25 (n = 385)	1.576 ± 0.26 (60)	0.763*
Eotaxin 2 (pg/mL)	3.03 ± 0.19 (n = 384)	3.03 ± 0.22 (n = 56)	0.937

*P* values were analyzed by Pearson's χ^2^ and Student's tests for categorical variables and continuous variables. *P values were analyzed by the Mann-Whitney *U* test. Serum cytokines levels are shown as log-transformed values. Values in bold indicate significant *P* ones.

EDN, eosinophil-derived neurotoxin; FEV1, forced expiratory volume in 1 s; IL-8, interleukin 8; MPO, myeloperoxidase; slgE, specific IgE; SPT, skin prick test; TGF-β1, transforming growth factor beta-1; WRS, work related symptom; yrs, years

**Table 2:** Comparation of clinical characteristics of exposed subjects according to the presence of WRS

	WRS+ (N = 70)	WRS- (N = 315)	*P* value
Age (yr)	36.21 ± 7.83	(312) 34.81 ± 7.79	0.227*
Gender (female, %)	36 (51.43%)	182/312 (58.33%)	0.292
Atopy (positive, %)	39/68 (57.35%)	93/306 (30.39%)	<0.001
Exposure duration (working years)	3.94 ± 3.14 (n = 61)	3.97 ± 3.56 (n = 300)	0.815
FEV1 (% predicted)	96.60 ± 9.87 (n = 69)	94.41 ± 13.14 (n = 310)	0.121*
SPT to wheat (positive, %)	12/69 (17.39%)	13/306 (4.25%)	<0.001
Serum total IgE (IU/L)	2.05 ± 0.67 (n = 69)	1.88 ± 0.63 (n = 308)	0.052
sIgE to Wheat (positive, %)	4/56 (6.78%)	18/289 (6.23%)	0.810
IL-8 (pg/mL)	2.19 ± 0.54 (n = 62)	2.14 ± 0.60 (n = 314)	0.526*
MPO (ng/mL)	2.12 ± 0.21 (n = 62)	2.07 ± 0.24 (n = 314)	0.120
TGF-b1 (pg/mL)	1.37 ± 0.16 (n = 70)	1.46 ± 0.11 (n = 315)	<0.001
EDN (ng/mL)	1.75 ± 0.26 (N = 70)	1.53 ± 0.23 (n = 315)	<0.001
Eotaxin 2 (pg/mL)	3.01 ± 0.19 (n = 69)	3.03 ± 0.19 (n = 315)	0.386

*P* values were analyzed by Pearson's χ^2^ and Student's tests for categorical variables and continuous variables. *P values were analyzed by the Mann-Whitney *U* test. Serum cytokines levels are shown as log-transformed values. Values in bold indicate significant *P* ones.

Abbreviations are described in Table 1

### Comparison of serum levels of epithelial-derived cytokines and EDN according to WRS

**Fig. 1:**
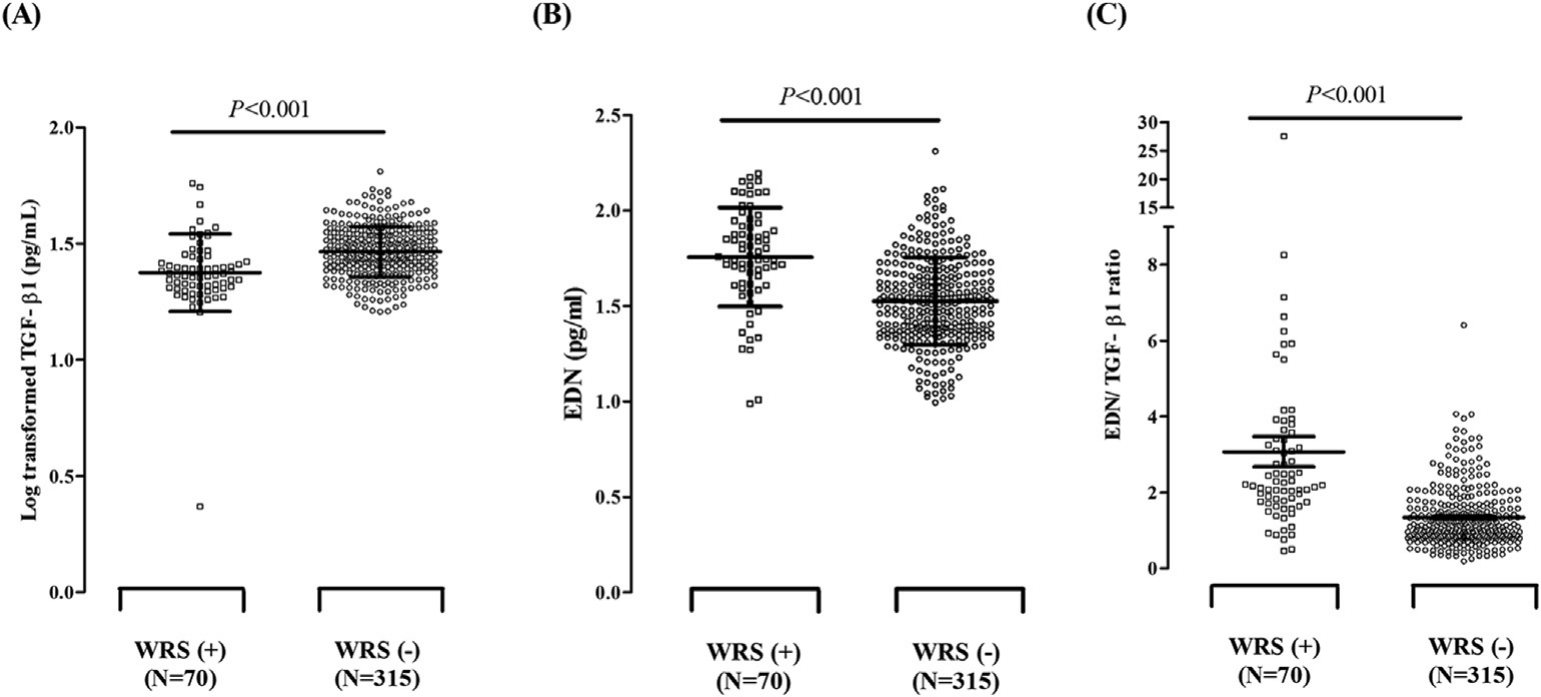
**Comparison of serum TGF-β1 (A), EDN (B) levels and the EDN/TGF-β1 ratio (C) between the WRS+ and WRS- groups.** Log-transformed data are presented as mean ± SD. *P* values were analyzed by the unpaired Student's *t*-test. EDN, eosinophil-derived neurotoxin; TGF-β1, transforming-growth factor β1; WRS, work-related symptoms

The mean exposure duration to wheat flour and total serum IgE level were not different between the WRS+ and WRS- groups. The serum EDN levels were significantly higher in the WRS+ group than in WRS- group, while the serum TGF-β1 levels were significantly lower in the WRS+ group than in the WRS- group (Fig. 1A and B, *P* < 0.001 for all). The exposure duration to wheat was divided according to exposure duration (<5 years; ≥5 years). Serum levels of TGF-β1 were not significantly different between these 2 groups, however, by stratification based on dust intensity, subjects with higher exposure intensity for more than 5 years tended to show lower levels of serum TGF-β1. (data not shown). When the ratio of EDN to TGF-β1 (EDN/TGF-β1) was calculated, it was significantly higher in the WRS+ group than the WRS- group (*P* < 0.001, Fig. 1C), which remained statistically significant after adjusting for age and sex by multivariate logistic regression analysis (data not shown); however, no difference in IL-8, MPO or eotaxin-2 levels was observed between the 2 groups. The serum EDN level was correlated with serum IL-8 (*r* = 0.264, *P* = 0.038), MPO (*r* = 0.457, *P* < 0.001) and eotaxin-2 levels (*r* = 0.301, *P* = 0.012) in the WRS+ group (data not shown) (see Table 2).

### Serum EDN and TGF-β1 levels for differentiating WRS+ and WRS- workers

**Fig. 2:**
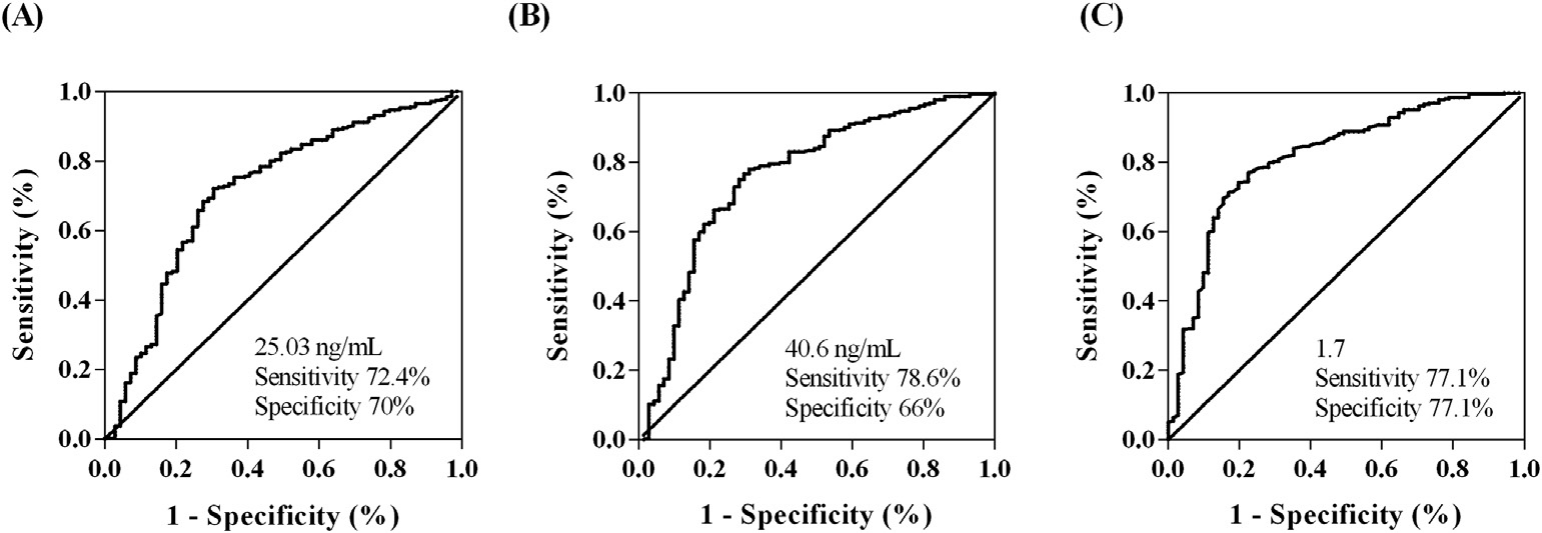
**Serum TGF-β1 (A) and EDN (B) levels and their ratio (C) for predicting the WRS+ group among exposed workers.** The receiver operating characteristic curve (ROC) analysis was performed to determine the cutoff value for predicting WRS. EDN, eosinophil-derived neurotoxin; TGF-β1, transforming-growth factor β1; WRS, work-related symptoms

The AUC value of serum EDN that predicts WRS+ workers was 0.759 (*P* < 0.001) with 78.6% sensitivity and 66% specificity at a cutoff value of 40.6 ng/ml. Although serum TGF-β1 levels were lower in the WRS+ group, the AUC of serum TGF-β1 that predicts WRS- workers was 0.719 (*P* < 0.001) at a cutoff value of 25.3 ng/ml with 72.4% sensitivity and 70% specificity as shown in Fig. 2 A and B. When the AUC value of the EDN/TGF-β1 ratio was applied, it increased to 0.814 at a cutoff value of 1.70 with 77.1% sensitivity and 77.1% specificity (Fig. 2C). Meanwhile, serum eotaxin-2 could not discriminate WRS+ in exposed subjects, with the AUC was 0.461 (data not shown).

### Changes in cytokine release from HAECs exposed to wheat

**Fig. 3:**
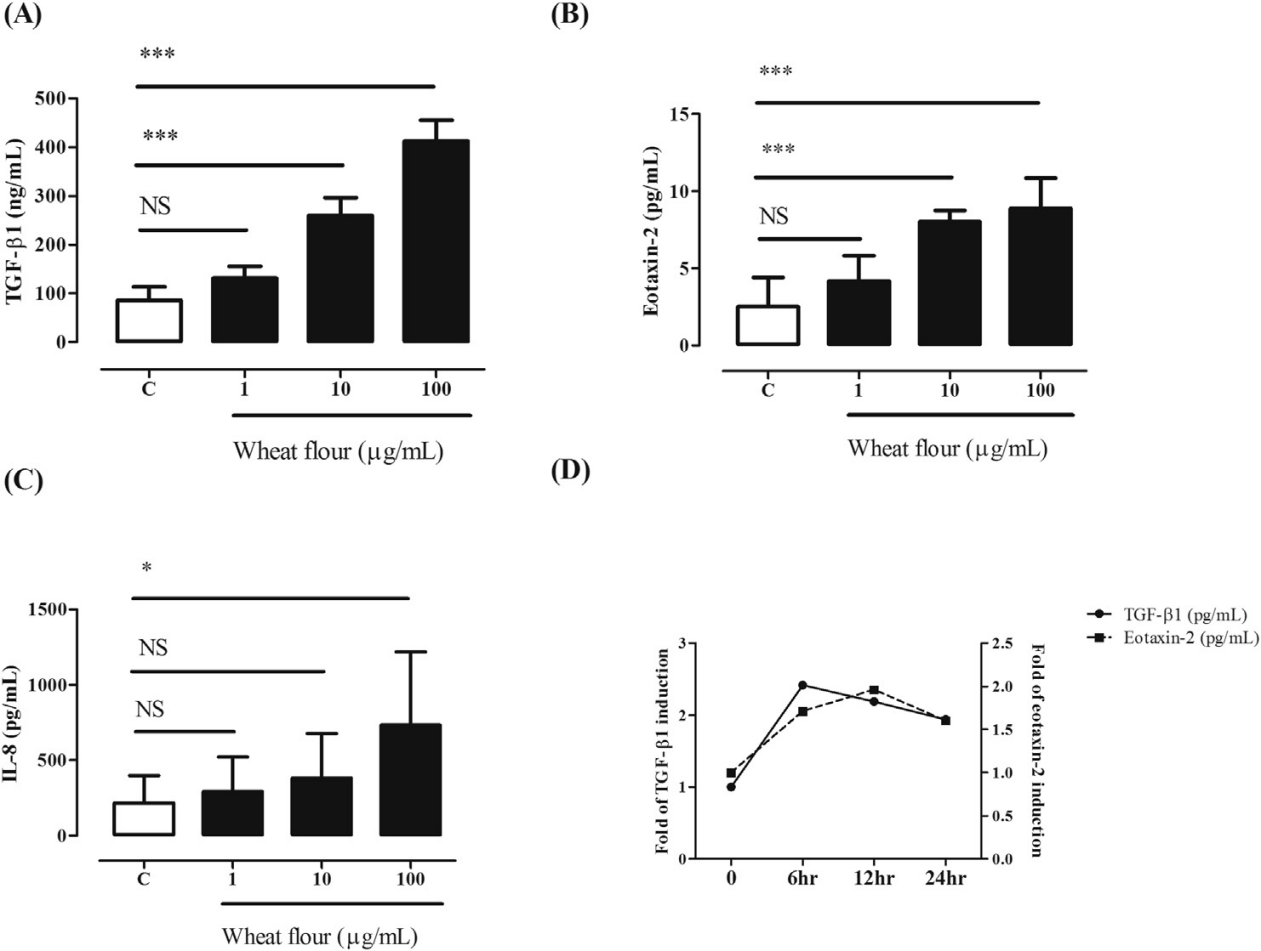
**Wheat-induced TGF-β1 (A), eotaxin2 (B) and IL-8 (C) productions from HAECs with their kinetic changes in a time-dependent manner**. *P* values were analyzed by one way ANOVA with Tukey post hoc test. *P < 0.05**,** **P < 0.01 and ***P < 0.001 compared between the indicated groups; NS, not significant. IL-8, interleukin-8; TGF-β1, transforming-growth factor β1

The optimal concentration of wheat antigens to stimulate HAECs was chosen based on the previous study and preliminary experiments.^14^ Wheat exposure increased the release of TGF-β1 and eotaxin-2 as well as IL-8 in a dose-dependent manner (*P <* 0.05, Fig. 3A–D). The TGF-β1 levels released were positively correlated with the levels of eotaxin-2 (*r* = 0.864, *P* < 0.001) and IL-8 (*r* = 0.618, *P* = 0.001) (data not shown). The TGF-β1 levels increased and peaked at 6 hours after wheat exposure (100 μg/mL). The eotaxin-2 levels increased significantly 12 hours after wheat exposure and tended to decrease until 24 hours (Fig. 3E).

### Effects of PBNs on TGF-β1 release from HAECs

**Fig. 4:**
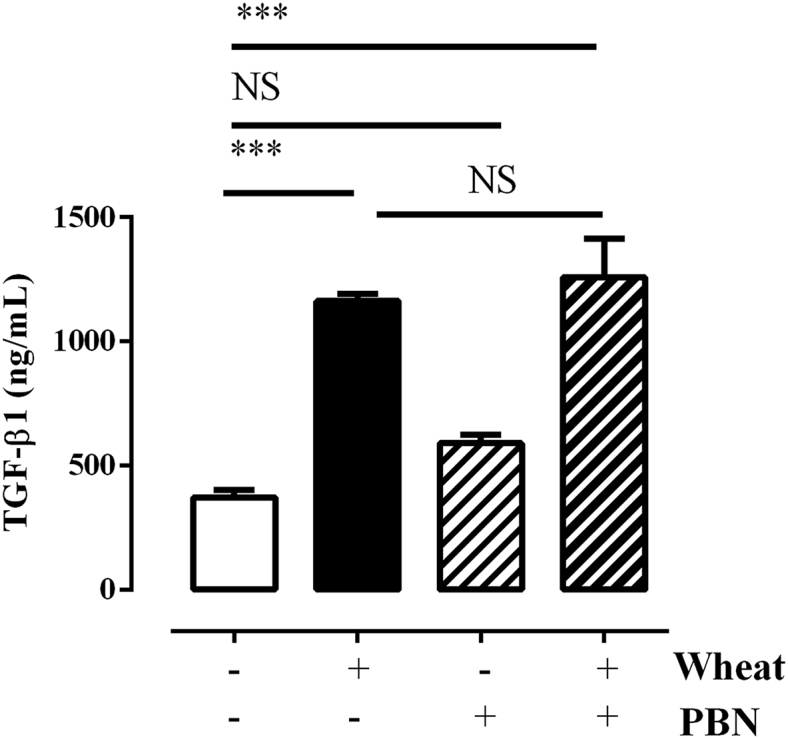
**Wheat-induced release of TGF-β1 from HAECs co-cultured with peripheral blood neutrophils** (**PBNs) of asthmatic patients.** A549 cells (1 × 10^5^) were seeded onto the lower chamber of Transwell system, stimulated with wheat flour extract (10 μg/mL). PBNs (5 × 10^5^ cells) were suspended onto the upper chamber of Transwell system and co-incubated with A549 cells for 48 h. *P* values were analyzed by one way ANOVA with Tukey *post hoc* test. ***P < 0.001 compared between the indicated groups; NS, not significant. TGF-β1, transforming-growth factor β1

As IL-8 and MPO levels were increased in exposed workers, HAECs were co-cultured with PBNs (ratio 1:5) obtained from asthmatic patients under the stimulation of wheat flour in order to investigate whether wheat antigen and PBNs lead to the exhaustion of TGF-β1 release. PBNs slightly triggered the release of TGF-β1 from HAECs; however, addition of wheat antigen induced a remarkable increase in TGF-β1 release. Thus, PBNs do not have any additive effect on TGF-β1 release from HAECs (Fig. 4).

## Discussion

There have been many efforts to elucidate the pathogenesis and predisposing factors for the development of WRS after wheat exposure,^1^, ^2^, ^10^, ^13^, ^20^, ^21^ since various innate/adaptive immune responses to wheat are involved in the developments of WRS.^15^ This study was in line with previous studies showing that Th2 response to wheat (positive SPT) and atopic status are associated with WRS. In addition, this study demonstrated that a significantly higher EDN level with a lower TGF-β1 level may be useful for differentiating between the WRS+ and WRS- groups among exposed bakery workers, which suggests that TGF-β1 derived from airway epithelial cells may contribute to develop type-2 airway inflammation, leading to development of WRS.

There is increasing evidence to demonstrate TGF-β1 as the key immunoregulatory cytokine of epithelial cells, which interacts with various immune cells in airway inflammation.^22^, ^23^, ^24^ Upon exposure to antigen, TGF-β1 may be derived from regulatory T (Treg) cells or HAECs with/without interacting with neutrophils.^22^, ^25^ TGF-β1 can modulate lineage differentiation of T cells depending on the milieu.^23^ HAEC-derived TGF-β1 is a cofactor of innate lymphoid cells to induce type-2 airway inflammation.^24^ However, the role of TGF- β1 in wheat-exposed workers has not yet been investigated, as wheat antigen contains several mediators such as bacteria and fungi as well as allergenic components to induce mixed immune responses in HAECs, unlikely to those of allergic asthmatic airway.^26^, ^27^ Type-2 immune responses with eosinophil activation are important pathogenic mechanisms of BA.^18^, ^28^ Activated eosinophils are major effector cells to develop asthma symptoms and to release EDN, which is a biomarker for eosinophil degranulation in asthmatics and wheezy infants.^29^, ^30^ Regarding the role of innate immune responses in bakery workers, increased levels of IL-8 and S100A8/S100A9 released from HAECs were noted in exposed workers,^17^ while *TLR4* variants at −2027 A > G and −1608 T > C were associated with WRS development.^13^ In the present study, a higher percentage of SPT+ responders and a higher EDN level were noted in the WRS+ group, indicating that type-2/IgE-mediated responses are involved in the development of WRS. In addition, a significantly lower TGF-β1 level was noted in the WRS+ group. *In vitro* experiments demonstrated that wheat exposure induced TGF-β1 release from HAECs initially. TGF-β1 (mainly derived from Treg cells) inhibits Th2-cell differentiation and suppressed Th2 responses in asthmatic airway.^23^ Although little is known about the function of Treg cells in wheat-exposed bakery workers, there have been a few studies demonstrating the suppressive function of gluten-specific Treg cells after wheat exposure.^31^ Endotoxins, as well as IL-5, existing in the bakery workplace  could induce eosinophil degranulation,^32^ suggesting that innate immune responses with epithelial activation are involved in the development of WRS in bakery workers. TGF-β1 from HAECs may contribute to develop type-2 airway inflammation in exposed workers.

Integrated neutrophilic/eosinophilic inflammation has been documented in the pathogenesis of WRS in bakery workers. While most studies indicated neutrophil activation, a few demonstrated the association of eosinophil degranulation in BA.^13^, ^14^, ^21^, ^28^ The recent review showed close interplay between eosinophils and epithelial cells/neutrophils in asthmatic airways.^33^ The present study demonstrated higher serum EDN levels in the WRS+ group than in the WRS- group. Serum EDN levels correlated with neutrophil-associated mediators (IL-8 and MPO). It is suggested that the elevation in EDN levels could be the consequence of type-2 responses^34^ or the interaction between neutrophils and eosinophils after wheat exposure. IL-8-induced neutrophil migration enhances the recruitment of eosinophils through the epithelial layer.^35^, ^36^ The endotoxin component in wheat flour can activate reactive oxygen species (ROS) production from neutrophils.^37^ ROS injury can form eosinophil extracellular traps, which releases eosinophil granules including EDN.^32^ In addition, wheat exposure stimulated HAECs to release eotaxin-2, an eosinophil chemotactic cytokine,^38^ thus recruiting more eosinophils to the airway. Subsequently, released EDN can damage tracheal epithelial cells, enhancing inflammation and remodeling.^39^ Taken together, Th2-derived eosinophil activation and the cross-talk between neutrophils and eosinophils play a crucial role in initiating EDN release from eosinophils, which trigger the development of WRS.

The diagnosis of BA includes self-reported symptoms, sensitization results (SPT and serum specific IgE to wheat antigen), and the specific bronchoprovocation test with wheat flour allergen.^40^ However, sensitization results may be false-negative with different extracts from different companies.^41^, ^42^ Less than 20% of WRS+ subjects in our study reported positive SPT responses to wheat, which is lower than that of the previous study.^41^ The bronchoprovocation test is the gold standard despite the risk in strongly sensitized bakers.^20^, ^43^, ^44^ Therefore, it is necessary to develop useful biomarkers for predicting WRS among bakery workers. In the present study, based on ROC curves, the increased level of serum EDN (AUC = 0.759, sensitivity/specificity 78.6%/66%) and decreased level of serum TGF-β1 (AUC = 0.719, sensitivity/specificity 72.4%/70%) may help differentiate the WRS+ group from the WRS- group. The ratio of EDN/TGF-β1 enhances chances of discriminating between the 2 groups (AUC = 0.814, sensitivity/specificity 77.1%/77.1%). Thus, both serum levels of TGF-β1 and EDN may be applied as non-invasive serum biomarkers for the early detection of WRS.

This study has 2 limitations. First, we did not achieve sputum or peripheral neutrophil count as well as eosinophil counts to prove their roles in the pathogenesis of WRS. Secondly, further decrease of TGF-β1 was not observed when PBNs were co-cultured with HAECs; we did not observe Treg responses in response to wheat antigen in the study subjects; therefore, we could not confirm mechanisms through which TGF-β1 is down-regulated. However, wheat exposure has been found to dysregulate the function of wheat-specific Treg subset,^31^ thereby affecting its down-stream pathways, and down-regulate TGF-β1 production.

## Conclusions

In conclusion, wheat exposure may induce the down-regulation of TGF-β1 in HAECs, which subsequently enhance type-2 airway inflammation; interactions between innate immune responses and activated neutrophils/eosinophils may amplify eosinophil degranulation, contributing to the development of WRS. Serum EDN and TGF-β1 may be potential serum biomarkers for predicting the development of WRS in occupationally exposed bakery workers. Further studies on the effect of the epithelial cell-TGF-β1-Treg axis on eosinophils are necessary in bakery workers.

## Consent for publication

Not applicable.

## Funding

This study was supported by a grant from the Korean Health Technology R&D Project, Ministry of Health& Welfare, South Korea (HI14C2628).

## Declaration of interest

The authors report no competing interests.

## Ethics statements

The study was approved by Ajou University Institutional Board. (AJIRB-GEN-SMP-13-108) and was carried out in accordance with the Helsinki declaration of the World Medical Association. All participants provided informed written consent to participate in the study.

## Authors’ contribution

Ulambayar B and Yang EM measured the cytokines in serum from patients. Trinh HKT and Cao TBT performed the *in vitro* studies. Lee SH collected clinical data from patients. Trinh HKT, Ulambayar B, and Cao TBT performed statistical analysis. Trinh HKT and Ulambayar B wrote the manuscript. Park HS conceived, designed the study, reviewed the manuscript and supervised the whole process.
