# Supplementary material for: Transforming growth factor-β1 and eosinophil-derived neurotoxins contribute to the development of work-related respiratory symptoms in bakery workers
Source: World Allergy Organ J. 2019 Oct 1;12(9):100058. doi: 10.1016/j.waojou.2019.100058 (PMC6796779; doi:10.1016/j.waojou.2019.100058)
Supplement: Multimedia component 1 [file mmc1.docx]

**SUPPLEMENTARY MATERIALS AND METHODS**

**Preparation of wheat flour extract and SDS-PAGE**

Wheat flour extract was prepared in sterile distilled water (1:10 wt/vol) at 4°C overnight, followed by centrifugation at 3000 rpm for 10 minutes. The supernatants were collected and filtered by syringe filter (0.22-μm pore size; Merck Millipore Ltd, Darmstadt, Germany). Filtered extract was concentrated using a Vivaspin 20-ml tube (10,000 MWCO; Sartorius Lab Intruments GmbH, Gowttingen, Germany) and protein concentration was measured by Bradford assay. Then, 20 μg of exracted wheat proteins were separated by SDS-PAGE in reducing and non-reducing condition on 12% acrylamide gel and stained with Coomassie blue for total protein analysis. Prepared extract was stored at －20℃ until used for *in vitro* experiments.

**Cell viability of HAECs after exposure to wheat**

Cells (2x10^4^) were seeded onto 96-well plates. To check for the toxicities of wheat flour extract on HAECs, different concentrations were introduced to HAECs and incubated for 24 hours in serum-free RPMI. Then, supernatants were collected, and cells were washed and re-suspended in serum and phenol-free RPMI. Cell viability was checked using the Cell Counting Kit-8 (Dojindo Molecular Technologies, Inc, Rockville, MD, USA) according to the manufacturer’s recommendation. The percentages of viable cells were normalized to the control wells.

**Table S1. Serum cytokine levels according to the results of sink prick test (SPT) to wheat and atopic status in exposed subjects.**

|  | **SPT+ (n=25)** | **SPT- (n=350)** | ***P* value** | **Atopy (n=132)** | **Non-atopy (n=242)** | ***P* value** |
| --- | --- | --- | --- | --- | --- | --- |
| IL-8 (pg/mL) | 2.15±0.58 | 2.14±0.58 | 0.987^*^ | 2.17±0.27 | 2.13±0.59 | 0.566^*^ |
| MPO (ng/mL) | 2.15±0.27 | 2.07±0.24 | 0.129 | 2.10±0.21 | 2.05±0.25 | **0.045** |
| TGF-β1 (pg/mL) | 1.42±0.25 | 1.45±0.11 | 0.591 | 1.44±0.15 | 1.45±0.11 | 0.476 |
| Eotaxin-2 (pg/mL) | 3.08±0.18 | 3.02±0.18 | 0.096 | 3.02±0.18 | 3.03±0.18 | 0.902 |
| EDN (ng/mL) | 1.70±0.27 | 1.55±0.24 | **0.004** | 1.64±0.24 | 1.40±0.96 | **<0.001** |

*P* values were analyzed Student’s *t* test for continuous variables. ^*^*P* values were analyzed by the Mann-Whitney *U* test. Serum cytokines levels are shown as log-transformed values. Values in bold indicate significant *P* values. EDN, eosinophil-derived neurotoxin; IL-8, interleukin 8; MPO, myeloperoxidase; SPT, skin prick test; TGF-β1, transforming growth factor β-1.

| **Table S2. Binary logistic regression analysis model for predicting development of WRS in exposed subjects** | | |
| --- | --- | --- |
|  | AOR (95% CI) | *P* value |
| TGF-β1 | 0.001 (0.000-0.017) | **<0.001** |
| EDN | 54.3 (15.8-186.9) | **<0.001** |
| EDN/TGF-β1 | 2.67 (1.43-4.95) | **0.002** |
| EDN, eosinophil-derived neurotoxins; TGF-β1, transforming growth factor β1. Values were log transformed for binary logistic regression analysis | | |

**SUPPLEMENTARY FIGURE LEGENDS**

**Fig. S1. Composition of wheat flour extract and toxicity of wheat flour on epithelial cells.** (A) 20 μg of protein were loaded onto SDS-PAGE and stained with Coomassie blue to visualize protein bands. Proteins were analyzed in 2 conditions: non-reduced (NR) and reduced (R). (B) Viability of A549 cells stimulated with wheat flour extract.
